# Predicting treatment response and clinicopathological findings in lupus nephritis with urine epidermal growth factor, monocyte chemoattractant protein-1 or their ratios

**DOI:** 10.1371/journal.pone.0263778

**Published:** 2022-03-10

**Authors:** Pintip Ngamjanyaporn, Suchin Worawichawong, Prapaporn Pisitkun, Khantong Khiewngam, Surasak Kantachuvesiri, Arkom Nongnuch, Montira Assanatham, Nuankanya Sathirapongsasuti, Chagriya Kitiyakara

**Affiliations:** 1 Faculty of Medicine Ramathibodi Hospital, Department of Medicine, Mahidol University, Bangkok, Thailand; 2 Faculty of Medicine Ramathibodi Hospital, Department of Pathology, Mahidol University, Bangkok, Thailand; 3 Faculty of Medicine Ramathibodi Hospital, Section of Translational Medicine, Mahidol University, Bangkok, Thailand; University of KwaZulu-Natal, SOUTH AFRICA

## Abstract

**Introduction:**

There is a need for sensitive and specific biomarkers to predict kidney damage and therapeutic response in lupus nephritis (LN). Monocyte chemoattractant protein-1 (MCP-1) and epidermal growth factor (EGF) are cytokines with divergent roles. EGF or EGF/MCP1 ratio have been shown to correlate with prognosis in primary glomerulonephritis, but there is limited information in lupus nephritis (LN). This study evaluated the roles of MCP-1, EGF or their ratio as biomarkers of histopathology and response to treatment in LN.

**Methods:**

This was a cross-sectional and observational study. Baseline urine MCP-1 and EGF levels in systemic lupus erythematosus (SLE) patients and controls (total n = 101) were compared, and levels were correlated with clinicopathological findings and subsequent response to treatment.

**Results:**

MCP-1 was higher in active LN (n = 69) compared to other SLE groups and controls, whereas EGF was not different. MCP-1 correlated with disease activity (proteinuria, renal SLEDAI, classes III/IV/V, and high activity index.) By contrast, EGF correlated with eGFR, but not with proteinuria, activity index, or class III/IV/V. MCP-1 was higher, and EGF was lower in high chronicity index. EGF/MCP-1 decreased with greater clinicopathological severity. In a subgroup with proliferative LN who completed six months of induction therapy (n = 41), EGF at baseline was lower in non-responders compared to responders, whereas MCP-1 was similar. By multivariable analysis, baseline EGF was independently associated with subsequent treatment response. Area under the curve for EGF to predict response was 0.80 (0.66–0.95). EGF ≥ 65.6 ng/ mgCr demonstrated 85% sensitivity and 71% specificity for response. EGF/MCP-1 did not improve the prediction for response compared to EGF alone.

**Conclusion:**

MCP-1 increased with disease activity, whereas EGF decreased with low GFR and chronic damage. Urine EGF may be a promising biomarker to predict therapeutic response in LN. EGF/MCP-1 did not improve the prediction of response.

## Introduction

Lupus nephritis (LN) is the most common severe manifestation of systemic lupus erythematosus (SLE) with a mortality rate of up to 25% in 5 years [1,2]. Appropriate immunosuppressive therapy improves the prognosis of LN, but up to 30% of patients may progress to kidney failure. Achieving a clinical response to induction treatment is critical to preserving long-term kidney health [3], but the high dose immunosuppressive regimens required may have deleterious side-effects [1,2], Predictors that can identify responders may enable the targeting of more aggressive therapy to those less likely to respond. Kidney biopsy is the gold standard for assessing the severity of LN. However, it is not feasible to perform repeated biopsies to follow disease progression as they carry some risks. Existing clinical and laboratory parameters do not reliably predict the renal histopathology or outcome [4,5]. Hence, there is still a need for developing novel biomarkers to monitor therapy in LN.

The severity of the kidney lesions and the response to therapy in LN are likely to depend on the balance between protective growth factors versus pro-inflammatory cytokines [2]. Monocyte chemoattractant protein-1 (MCP-1) acts as a chemokine promoting the migration of leucocytes into the kidneys [6]. In LN, correlations have been observed between urinary MCP-1 levels and disease severity [2]. However, there is still conflicting data on the role of MCP-1 in predicting response to therapy [7–9]. Because of the heterogeneity of LN, using one biomarker may not have satisfactory specificity and sensitivity. Combinations of MCP-1 with other biomarkers may increase its predictive value.

Messenger RNA profile studies from kidney disease patients identified epidermal growth factor (EGF) as a peptide growth factor that correlated with the estimated glomerular filtration rate [10]. In rodents, kidney injury has been shown to lower EGF [11]. Chronic kidney disease is associated with reduced urine EGF [12–14], and decreased urine EGF levels predicted response to steroid therapy in primary glomerulonephritis [15]. EGF1 and MCP-1 may have opposing roles in determining disease activity and treatment response of LN. EGF/MCP-1 ratio may predict adverse kidney function or the severity of the renal histopathology [16]. EGF/MCP-1 ratio may be superior to EGF or MCP-1 alone in predicting outcome in IgA nephropathy [12]. Currently, the role of EGF MCP1-1 ratio in predicting kidney pathology or response to treatment in LN is unknown. Both urine MCP-1 and EGF reflect local production and may be helpful in monitoring kidney injury in the setting of systemic manifestations of SLE. In this study, we investigated the hypothesis that EGF, MCP-1, or their ratio correlates with the clinicopathological parameters and predicts response to treatment in LN.

## Methods

### Study subjects

This was a single-center, cross-sectional, observational cohort study of patients with SLE age 15–70 and healthy controls conducted at Ramathibodi Hospital, a tertiary center in Bangkok, Thailand. Subjects were recruited from Jan 2015 until December 2018. We performed this study conforming to the World Medical Association Declaration of Helsinki. This investigation was endorsed by the Faculty of Medicine, Ramathibodi Hospital Ethics Committee (ID 2021/138). Written informed consent was provided by the patients. Subjects with pregnancy or infections were excluded.

SLE patients who fulfilled the 1997 American College of Rheumatology criteria were enrolled [17]. The Systemic Lupus Erythematosus Disease Activity Index (SLEDAI) and renal SLEDIA (rSLEDAI) scores were calculated to evaluate overall and renal disease activity [18]. Patients with a SLEDAI score >4 were considered as active SLE otherwise they were classified as inactive SLE (INA). Active SLE patients with rSLEDAI score >0 were classified as active Lupus nephritis (ALN), and the remaining in the active non-nephritis (ANR) group.

Healthy controls (CON) were subjects without underlying chronic conditions requiring regular medications with normal blood pressure and blood sugar and urinalysis with similar age and sex ratio the ALN group.

Fasting blood and second morning urine were collected [19]. In ALN patients, urine was collected on the day or within 2 weeks before the biopsy. Thirty milliliters of urine was centrifuged at 4,000 rpm for 10 minutes, and then the supernatant was stored at -80°C.

### Kidney histopathology

Kidney biopsies were performed according to clinical indications in patients with proteinuria≥ 0.5g/24 hours [20]. Glyo-Fixx (Thermo scientific, USA) was used to fix the tissues, which were then embedded in paraffin, and cut into sections (2 μm) for light microscopy, immunofluorescence, and electron microscopy. A nephropathologist, without knowledge of the laboratory data, classified and calculated the activity index (AI) scores and the chronicity index (CI) scores according to the International Society of Nephrology/Renal Pathology Society Classification [21]. Patients with combined III or IV and V were classified as class III or IV. Adverse prognosis was indicated as AI scores of ≥7 or CI scores of ≥4 [22].

### Laboratory and urine cytokine measurements

We assayed creatinine in blood and urine using the Enzymatic method (Dimension ExL analyzer, Siemens Healthcare Diagnostics, Newark, DE USA) and used the CKD-EPI equation for non-blacks to calculate the estimated glomerular filtration rate (eGFR; mL/min/1.73m^2^) [23]. Urine protein was assessed by modified pyrogallol red molybdate.

ELISA kits for MCP-1 and EGF (Quantikine ELISA Immunoassay R&D systems) were used with details similar to our previous publication [15]. CV% were: MCP-1-2.6% and EGF-3.2%. Results were expressed as ng per mg of creatinine (UMCP-1 and UEGF) or as a ratio.

### Renal outcome

Patients were treated according to physician preference based on published guidelines [20]. Patients with class III/IV received induction with high/ low dose intravenous cyclophosphamide, mycophenolate [24,25] or mutitargetted regimen [26]. The outcome was combined renal response to therapy (completed remission or partial remission) assessed at six months using KDIGO criteria where complete remission was defined by proteinuria less than 0.5 gram per day, and partial remission by decreased proteinuria more than 50% and less than 3g/24 hours from baseline with stable renal function [27]. In our study, urine protein creatinine ratio (UPCR) was used instead of 24 hours protein collection.

### Statistics

Data are reported as median (P25^th^, P75^th^), mean ± standard deviation, or frequency (percentage), as appropriate. We used independent t-test or Mann-Whitney test or Chi-square test to compare two groups. The analysis of variance or Kruskal–Wallis tests were used to compare multiple subgroups. Pairwise post-hoc comparisons were performed using the Dunn-Bonferroni procedure. We used Spearman’s test to assess the correlations between biomarker levels and renal function. The association of the baseline cytokines to the therapeutic outcome was evaluated by multivariable logistic regression. We constructed receiver-operating characteristic (ROC) curves to determine the areas under the curves (AUC (95% CI)) of MCP-1, EGF, and EGF/MCP-1 for histology features or response to therapy. Values with the best sensitivity and specificity for each cytokine were used to categorize patients into low or high cytokine groups. For class, activity, and chronicity index, the inverse of EGF or MCP-1/EGF were used to evaluate AUC. SPSS version 23 was used for analyses. Two-tailed p was <0.05 was considered significant.

## Results

### Patient characteristics

A total of 101 SLE patients (active Lupus nephritis (ALN, n = 69); active non-nephritis (ANR, n = 21); inactive SLE (INA, n = 21)) and 11 healthy controls (CON) were recruited. Baseline characteristics are shown in **Table 1**. Patients with active LN were younger, had a higher proportion of females, higher systolic blood pressure, serum creatinine, proteinuria, renal SLEDAI, SLEDAI, and lower hemoglobin than patients with inactive SLE or active non-renal lupus.

**Table 1:** Baseline characteristics of SLE patients and healthy controls.

Characteristics	Active LN (n = 69)	Inactive SLE (n = 21)	Active non-renal lupus (n = 11)	Healthy controls (n = 11)	p
Age (years)	32 ±11	42 ±14 ^a^	36 ±15	42 ± 7 ^a^	0.001
Female (%)	65 (94.2%)	19 (90.5%)	8 (72%) ^a^	10 (90%)	<0.001
SLE duration (years)	6 (2, 11)	6 (4.5, 14.5)	2 (0, 9)	-	
Body mass index (kg/m2)	23.2 ±4.5	22.1 ±7.1	23.1 ±3.4	23.0 ± 3.9	0.482
Systolic blood pressure (mmHg)	130± 15	112± 20 ^a^	119 ± 17	110 ± 14 ^a^	<0.001
Diastolic blood pressure (mmHg)	79 ± 12	70 ±6	74±10.35	69 ±12	0.101
Hemoglobin (g/dl)	11.3 ±1.3	12.4±1.0 ^a^	11.6 ±0.94 ^b^	13.0 ±0.9 ^a^^, c^	<0.001
Serum Creatinine (mg/dl)	1.07 ± 0.75	0.69 ±0.14^a^	1.08 ±0.99	0.68 ±0.15 ^a^	0.134
eGFR (ml/min/1.73m^2^)	89 ±37	105 ±13	96 ±35	107 ±13.6	0.522
Proteinuria (g/g Creatinine)	3.36 (3.66)	0.22 (0.12) ^a^	0.24 (0.13) ^a^	0.12 (0.06) ^a^	<0.001
Renal SLEDAI	8 (4, 12)	0 (0, 0) ^a^	0 (max 0) ^a^^,^^b^	-	<0.001
SLEDAI	10 (6,12)	0 (0, 0)^a^	2 (2,7)^a^^,^^b^	-	<0.001

Data as mean ± SD; median (25, 75 percentile); LN, lupus nephritis; eGFR, estimated glomerular filtration rate.

^a^ = p < 0.05 vs active lupus nephritis.

^b^ = p < 0.05 vs inactive SLE.

^c^ = p < 0.05 vs active non-renal lupus.

### Urine cytokines levels in all SLE patients and controls

**Fig 1:**
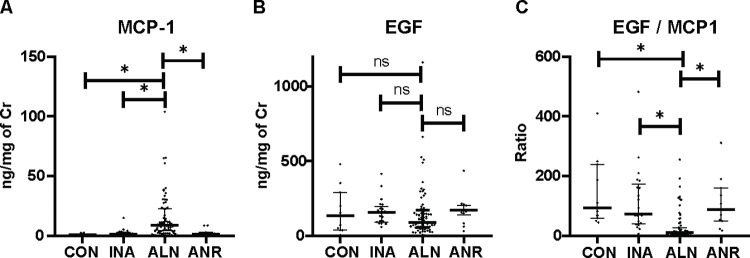
Urine cytokines in SLE patients and healthy controls. a) MCP-1, b) EGF and c) EGF/MCP-1. Active lupus nephritis (ALN), n = 69; Active non-renal (ANR), n = 21; Inactive SLE (INA), n = 21; and healthy controls (CON), n = 11). *p<0.05. Cr, creatinine.

MCP-1 was significantly higher in active lupus nephritis (ALN) compared to other groups (ALN; 9.0 (4.2, 22.8) vs ANR 1.4 (0.7, 3.1); INA 1.5 (1.0, 2.8); and CON 1.1 (0.6, 1.4); p < 0.001). No differences were observed between other groups **(Fig 1).** EGF/MCP-1 was significantly lower in the ALN compared to other groups (ALN, 11.0 (4.9, 27.2); ANR, 88.6 (50.7, 160.7); INA, 72.8 (42.9, 162.4) and CON, 93.5 (59.1, 239.4); p <0.001)). In contrast, EGF was not different between groups (ALN 90.9 (55.7, 163.5); ANR, 163.5 (83.8, 210.7); INA, 156.2 (93.2, 183.8); and CON 136.4 (38.3, 291.8); p = 0.12)

### Relationship of cytokines with clinical parameters

The relationship of cytokines with clinical characteristics at baseline was evaluated in all SLE patients (n = 101). Urine MCP-1 correlated with proteinuria, and tended to correlate inversely with GFR. **(S1 Table)** Urine EGF correlated with GFR, but not with proteinuria. EGF/MCP-1 ratio correlated with GFR, and inversely with proteinuria. Renal SLEDAI correlated with MCP1, inversely with EGF/MCP-1, but not with EGF.

### Urine cytokines and kidney histopathology

The relationship between urine cytokines and baseline clinicopathological features were evaluated in ALN (n = 69).

#### WHO class

**Fig 2:**
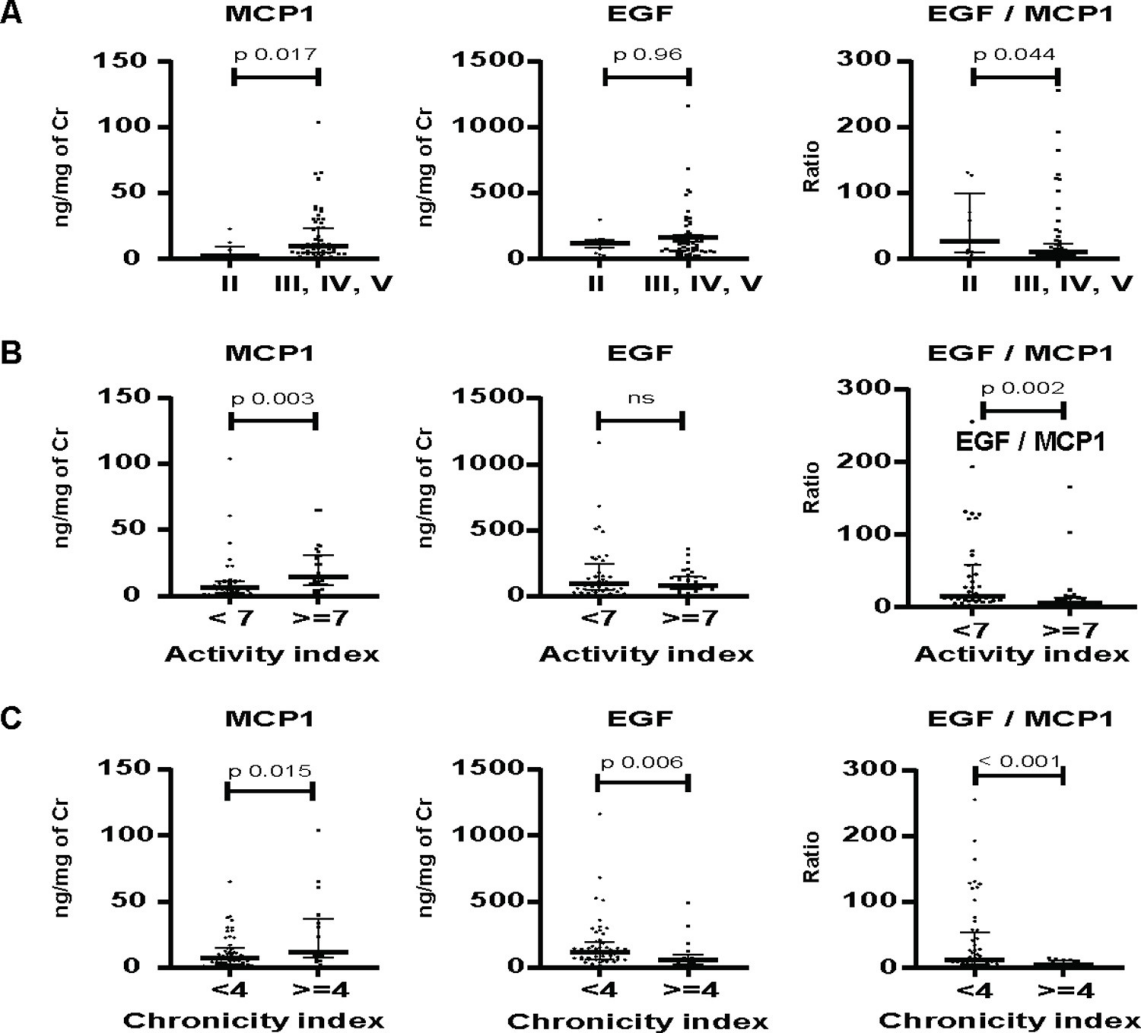
Urine cytokines levels and renal histology (n = 69). a) WHO Class: Class II versus Combined Classes III/IV/V; b) Activity index: (AI High (≥7) Versus Low (<7); c) Chronicity Index (CI): High ((≥7) versus Low (< 7). Cr, creatinine.

The WHO class were: II (n = 9), III (n = 25 (including 15 III+ V)), IV (n = 21 (including 7 with IV+V), V (n = 13), and VI (n = 1). To further evaluate the differences between class II and more active nephritis, we combined patients with class III, IV, V. **(Fig 2).** MCP-1 was significantly higher in the combined class (MCP1: Combined, 9.7 (4.9, 23.8) versus Class II 2.4 (1.7, 6.9), p = 0.017). EGF/MCP-1 was significantly lower in the combined class. (EGF/MCP1: Combined, 10.4 (4.0, 23.3) vs. Class II, 26.7 (11.4, 71.2), p = 0.044.) By contrast, EGF was not different between the 2 groups. (EGF: Combined, 90.9 (55.8, 186.8) vs. Class II, 117.3 (45.5, 1 51.9), p = 0.96).

The AUC to classify combined versus class II was: MCP-1 0.75 (0.56, 0.94). EGF or MCP-1/EGF had lower AUC: 1/EGF, 0.50 (0.30, 0.70); and MCP-1/EGF, 0.71 (0.55–0.87). MCP-1 ≥ 3.86 ng per mg Cr gave 86% sensitivity and 67% specificity to detect combined class.

#### Activity index

Overall, AI was 5 (0, 8). Forty (58%) patients had high AI (≥7) **(Fig 2)**. High AI group had higher MCP-1, lower EGF/MCP1, whereas EGF was not different. (MCP-1: High AI, 14.3 (8.5, 30.4) vs. Low AI, 6.5 (2.5, 11.0), p = 0.003. EGF/MCP-1: High AI, 5.3 (2.4, 12.8) vs. Low AI, 14.5 (8.4, 57.9), p = 0.002. EGF: High AI, 84.7 (60.0, 144.0) vs. Low AI, 97.6 (49.9, 224.8), p = 0.6.

The AUC for high AI were comparable between MCP-1 and MCP-1/EGF. (AUC: MCP1, 0.71(0.58, 0.84); MCP-1/EGF, 0.72 (0.59–0.84); 1/EGF, 0.53 (0.39–0.67). MCP-1 ≥ 8.45 ng per mg Cr gave 79% sensitivity and 65% specificity to detect high AI.

#### Chronicity index

Overall, CI was 2 (1, 3). **(Fig 2)**. Seventeen (25%) patients had high CI (≥4). High CI had higher MCP-1, and lower EGF and lower EGF/MCP1. (MCP-1: High CI, 11.5 (8.5, 33.6) vs. Low, 7.7 (3.2, 14.7), p = 0.015; EGF: High CI, 55.7 (26.6, 74.1) vs. Low CI, 120.2 (62.4, 191.6), p = 0.006; EGF/MCP-1, High CI, 4.8 (1.8, 11.5) vs. Low CI, 12.6 (6.5, 51.2), p<0.001.

AUC of high CI were comparable, but MCP-1/EGF tended to be better than either cytokine alone. (AUC: MCP1, 0.70 (0.56, 0.84); 1/EGF, 0.72 (0.56–0.81); MCP-1/EGF 0.78 (0.66, 0.90). MCP-1/EGF ≥ 0.08 gave 88% sensitivity and 50% specificity for high CI.

### Urine cytokines as predictors of treatment response

The relationship between baseline cytokine levels and response to induction treatment at 6 months in proliferative LN (Class III or IV). Of 46 patients, 41 (IV = 11, IV+V = 7, III = 10, III+V = 13) completed 6 months follow-up. The induction regimen were intravenous cyclophosphamide (n = 26), mycophenolate mofetil (n = 9) and combined immunosuppressive agents (n = 6). Of these patients, 27 (66%) responded to therapy (complete (n = 12), partial (n = 15)) at 6 months. Responders (RES) and non-responders (NR) had similar clinicopathological characteristics at baseline (**Table 2**).

**Table 2:** Baseline clinicopathological characteristics of active lupus class III or IV by response to induction therapy at 6 months (N = 41).

Parameters	Non- Responders (N = 14)	Responders (N = 27)	p
Age (years)	31 ±10	28 ±12	0.89
Females (%)	93%	100%	0.16
Disease duration (year)	3.5 (1, 15)	6 (2, 12)	0.54
IV Cyclophosphamide	11/14 (78.6%)	15/27 (55.6%)	0.15
Complement C3 (mcg/ml)	892 (478, 1140)	567 (365, 842)	0.21
Complement C4 (mcg/ml)	185 (70, 299)	97 (69, 231)	0.24
Proteinuria (g per g creatinine)	2.27 (1.18, 2.57)	2.92 (1.75, 4.48)	0.22
Glomerular filtration rate (ml/min/1.72m^2^)	75 ±35	95 ±40	0.10
SLEDAI	12 (8,12)	10 (6, 14)	0.96
Renal SLEDAI	8 (8,12)	8 (4, 12)	0.97
Class IV	6 (42%)	12 (44%)	0.92
Class V	7 (50%)	13 (48.2%)	0.91
Activity index	8 (5, 9)	8 (4, 10)	0.78
Chronicity index	2.5 (1, 3)	2 (1, 3)	0.80

Data as Median (25,75 th percentile) or Mean ± SD.

**Fig 3:**
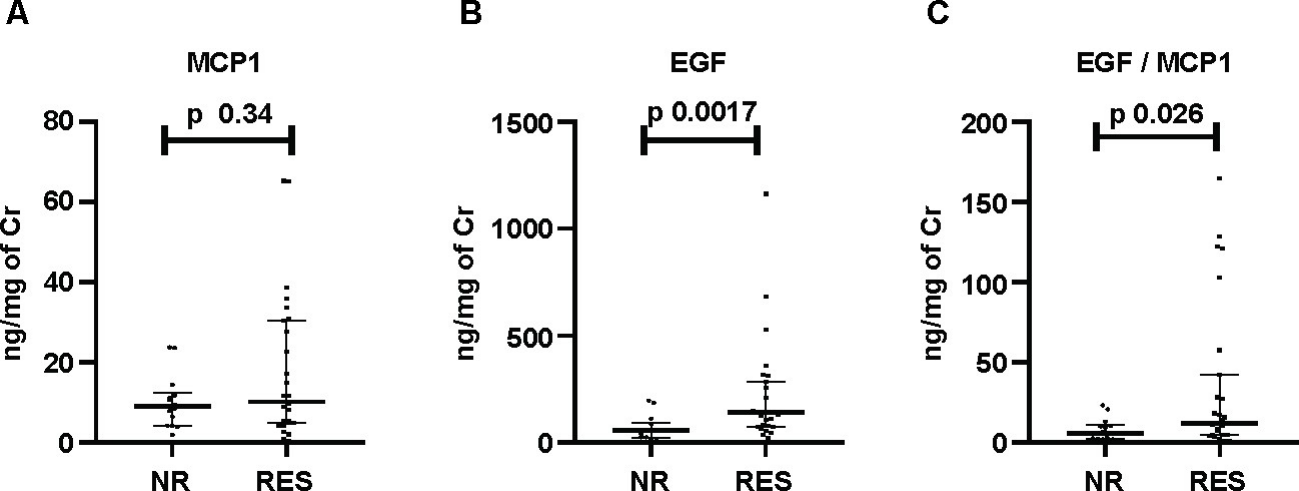
Urine cytokines levels A) MCP-1; B) EGF; C) EGF/MCP-1 for response to induction therapy at 6 months in proliferative nephritis (n = 41). Responders (RES), Non-responder (NR). Cr, Creatinine.

**Fig 3** shows that the urine EGF and EGF/MCP-1 at baseline were significantly lower in NR compared to RES (EGF: NR, 56.6 (23.8, 86.6) vs. RES, 141.5 (74.1, 283.5), p = 0.0017; EGF/MCP1: NR, 5.8 (2.1, 10.36) vs. RES, 11.9 (4.9, 42.1), p = 0.026. However, MCP-1 was not different (MCP-1: RES; 8.96 (4.21, 14.37) and NR 7.52 (2.15, 17.21), p = 0.34.

#### Multivariable analysis

**Fig 4:**
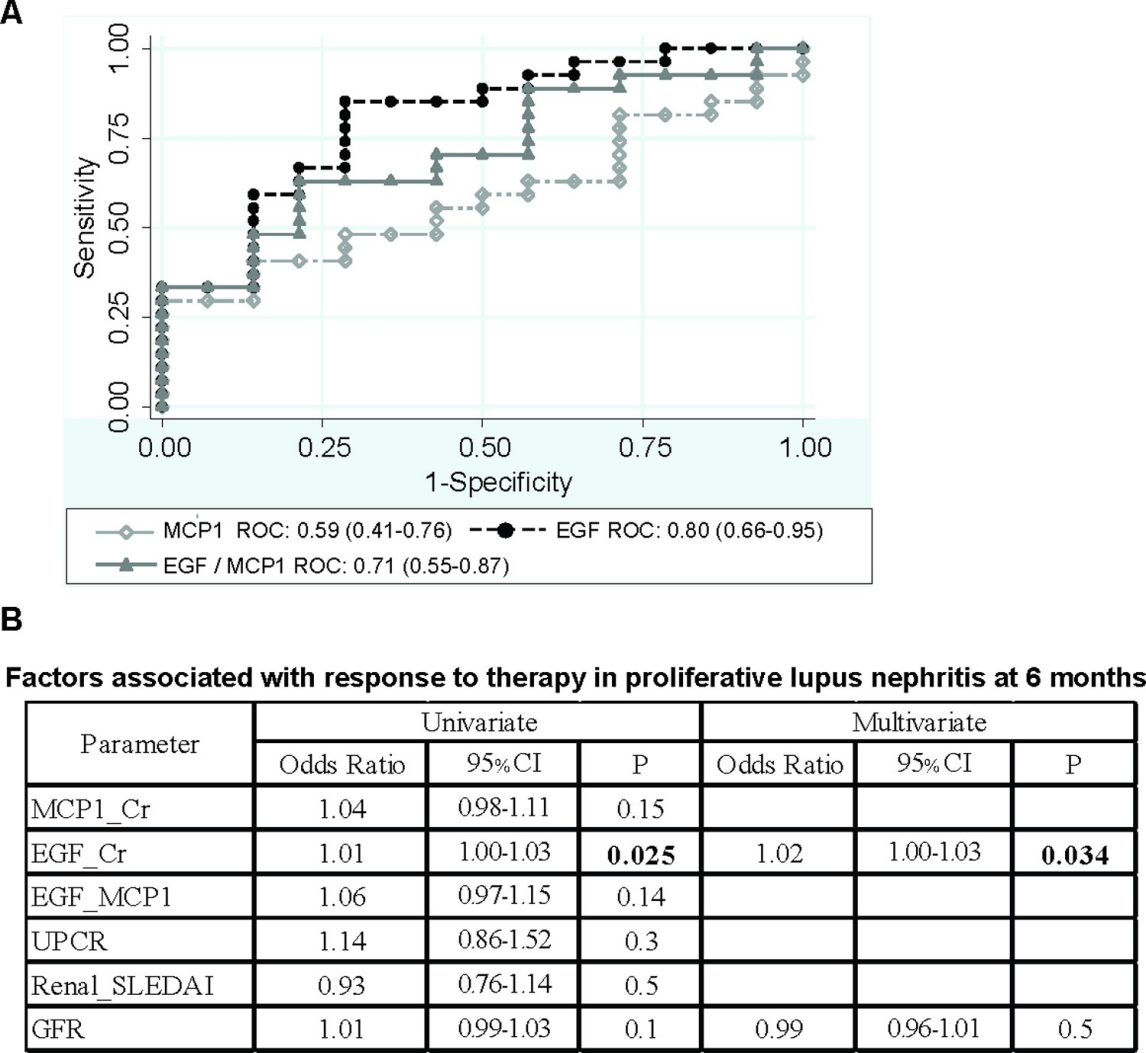
Urine cytokines as predictors for response to induction therapy at 6 months in proliferative nephritis (n = 41). A) Receiver operating curve to predict response. AUC (95% confidence interval); B) Univariate and multivariate analysis of factors to predict response.

By univariate analysis, EGF and eGFR at baseline correlated or tended to correlate with response to therapy **(Fig 4)**. By multivariate analysis, EGF was an independent predictor for response to therapy at 6 months.

#### ROC curve for predicting response at 6 months

 The AUC to predict response at 6 months for EGF was 0.80 (0.66–0.95) **(Fig 4).** The AUC for EGF/MCP1 and MCP1 were lower. EGF ≥ 65.6 ng/mgCr gave 85% sensitivity and 71% specificity for combined response at 6^th^ month.

## Discussion

The main new results of this study were that baseline levels of EGF was independently associated with response to therapy at 6 months and that the ratio of EGF/MCP-1 did not aid the forecast of the response to therapy when compared to EGF on its own despite being correlated with clinical and histology features. MCP-1 and EGF mostly showed divergent relationships with clinicopathological features and treatment response in LN. MCP-1 was markedly elevated active LN, but did not predict response. In contrast, EGF correlated with GFR and chronicity index, but was not increased in heavy proteinuria, high activity index or in class III/IV/V.

MCP-1 is produced by residential renal cells and leucocytes in response to immune complex deposits with a key role in the pathogenesis of nephritis [6,28]. Higher MCP-1 in active LN compared to active SLE patients without nephritis is consistent with previous data. [29] Earlier studies have shown that urinary MCP-1 was increased with higher renal SLEDAI, activity index and in classes III, IV and V [7,30–32], but the correlations between AI and CI with MCP-1 were not universal [7,33]. These differences may be due to variations in the numbers and severity of patients in each study.

EGF mediates cell growth and differentiation and promote cell survival through autocrine/paracrine signaling at the epidermal growth factor receptor [11]. EGF is mainly synthesized locally within the kidney as glomeruli and tubules express EGF protein and mRNA [10,34]. Earlier investigators have shown that there was a positive correlation between urine EGF with eGFR in primary GN [16], diabetic nephropathy [14], and an inverse correlation with the degree of tubulointerstitial chronicity in primary GN [16]. As far as we know, there has been only one study evaluating the role of EGF in LN. Mejia-Vilet et al evaluated the relationship of urinary EGF to renal histology and long-term change in GFR in US and Mexican patients with LN [35]. They found that EGF correlated with chronicity index and predicted long term GFR levels, but did not correlate with activity index. The authors did not investigate the role of EGF in predicting response to induction therapy. EGF has not been studied in other LN populations, including Asians whose disease may be more severe [36]. The results from our study of Thai patients provide additional evidence supporting the use of EGF as a universal biomarker of chronicity in different populations. We provided additional information by showing that protein excretion did not correlate with EGF, consistent with the notion that levels of EGF provides an assessment of tubular injury rather than glomerulitis. Moreover, we demonstrated that EGF independently predicted the response to induction treatment. In the US/Mexican cohort, patients with active LN had lower EGF than SLE patients without nephritis, whereas we did not observe any differences between groups. The differences in findings might be because the US/Mexican patients had reduced baseline eGFR, whereas our patients mostly had normal GFR.

Treatment response is one of the most important indicators of kidney survival in proliferative LN [1–3]. Currently, there are no definitive biomarkers for predicting response. Urine MCP-1 was found to be higher in responders in some [7,8] but not all studies [9]. In this study, MCP-1 levels were similar between responders and non-responders. To the best of our knowledge, this study is the first study to show that the response to induction therapy in was independently in LN was associated with reduced baseline levels of urine EGF. This is consistent with previous data in primary GN [15]. The mechanisms whereby low urine EGF could be linked to therapeutic response in LN are unclear. EGF may improve kidney recovery by stimulating regeneration of tubular cells, decreasing apoptosis and fibrogenic response to injury [34,37–39]. In proliferative LN, immune complex deposition leads to glomerular visceral epithelial cells (podocytes) damage and proteinuria. Recently, EGF has been shown to promote podocyte proliferation and re-expression of differentiation markers after exposure to high glucose concentrations [40]. Therefore, EGF could decrease proteinuria in LN by restoring glomerular function through its ability to repair podocytes. Since the generation of EGF is restricted mostly to the kidneys [12], levels of EGF in the urine may reflect local renal production. Consequently, LN patients with higher levels of EGF in the urine who responded to therapy may have less initial kidney injury and a higher ability to repair damaged podocytes and tubules.

Our results have several implications. Since EGF is mainly produced within the kidneys, systemic EGF is unlikely to add to urinary EGF levels. Unlike other potential biomarkers, EGF could be a useful biomarker for guiding treatment in LN that distinguishes kidney inflammation from systemic disease activity [35]. For instance, patients with reduced urinary levels of EGF, who may have an increased risk of poor response to standard induction, might be assigned to a more potent drug regimen right at the start. In addition, EGF might be a helpful biomarker of patient prognosis for stratifying patients in clinical trials of new drugs. Currently, there are no completely accurate biomarkers to predict response to induction treatment in LN. Therefore, the finding that EGF above 65.6 ng/mgCr had moderately good sensitivity and specificity for response could represent an advance in the field, although additional biomarkers may be needed to enhance the predictive performance. Earlier, prediction of outcome in IgA nephropathy [13] and obstructive uropathy was found to be better with EGF/MCP-1 ratio than either cytokine on its own [41]. This study showed no benefit in measuring EGF/MCP-1 in addition to EGF. Other biomarkers should be evaluated with EGF for any additive effects in predicting response. Lastly, our data support experimental studies that EGF may stimulate kidney repair. If confirmed in additional studies, enhancing EGF production may potentially lead to novel therapies in LN.

There are several limitations to this study. We only studied a limited number of patients from a single-center and measured urine cytokines levels only once at baseline. Using spot urine instead of 24-hour urine protein may cause some errors. Performing a 24-hour urine collection was not convenient for our patients. A second-morning urine sample has good correlations with 24 urine protein in lupus patients and is more practical for patients traveling from home for admissions for biopsies than first-morning urine [19]. Patients were treated according to clinician preferences based on published guidelines rather than a protocolized regimen [24,25]. Although we showed the ability of high EGF to predict outcome, a larger study using standardized treatment protocols with longer follow-up duration and repeated EGF measurements would be needed to assess the full benefit of EGF monitoring in the clinical management of LN. Finally, although the cost of the ELISA test for EGF is comparable to other standard tests used to evaluate LN activity, the cost-effectiveness of EGF measurement in improving patient outcome should also be demonstrated before the use of EGF can be routinely recommended.

## Conclusion

MCP-1 correlated with active disease and proteinuria, while EGF correlated with GFR and chronicity. The novel finding that urine EGF may be a promising candidate biomarker to predict response to therapy in LN could support its greater use in larger trials and ultimately in clinical practice to guide treatment decisions. While the ratio of EGF to MCP-1 correlated with clinicopathological parameters, the ratio did not predict response. Future studies to evaluate the role of EGF alone or in combination with other biomarkers should be considered.

## Supporting information

S1 Table Correlations of urine biomarkers with clinical parameters in all SLE patients (n = 101). (DOCX)

S1 File (XLSX)
