# Supplementary material for: Predicting treatment response and clinicopathological findings in lupus nephritis with urine epidermal growth factor, monocyte chemoattractant protein-1 or their ratios
Source: PLoS One. 2022 Mar 10;17(3):e0263778. doi: 10.1371/journal.pone.0263778 (PMC8912200; doi:10.1371/journal.pone.0263778)
Supplement: S1 Table — (DOCX) [file pone.0263778.s001.docx]

**Table S1: Correlations of urine biomarkers with clinical parameters in all SLE patients (n=101)**

|  | MCP-1/cr | EGF/cr | EGF/MCP-1 |
| --- | --- | --- | --- |
| eGFR | -0.19  (0.051) | 0.50  (<0.001) | 0.94  (<0.001) |
| Proteinuria | 0.72  (<0.001) | -0.09  (0.354) | -0.36  (<0.001) |
| RENAL SLEDAI | 0.34  (0.006) | -0.13  (0.212) | -0.49  (<0.001) |
